# Research on Measurement Principle and Key Measuring Devices of Pressure Change Rate for Electronically Controlled Pneumatic Brake of Commercial Vehicle Based on Poiseuille’s Law

**DOI:** 10.3390/s22083023

**Published:** 2022-04-14

**Authors:** Jian Hu, Min Yan, Rui Yang, Fan Yang, Gangyan Li

**Affiliations:** School of Mechanical and Electronic Engineering, Wuhan University of Technology, 122 Luoshi Road, Wuhan 430070, China; 271430@whut.edu.cn (M.Y.); yangrui_zw@163.com (R.Y.); yang_fan@whut.edu.cn (F.Y.)

**Keywords:** commercial vehicle, brake pressure change rate, measurement principle, isothermal container, laminar flow resistance tube

## Abstract

For intelligence brakes in the electronic pneumatic brake system of commercial vehicles, the pressure change rate is used as the key control parameter and evaluation index. This can improve the brake safety, stability, and ride comfort of the vehicle. The real-time detection of the brake pressure change rate for commercial vehicles is the premise for realizing the accurate control of brake pressure change rate. Based on Poiseuille’s law, an efficient measurement method of brake pressure change rate is proposed for commercial vehicles, and a new measuring device with an isothermal container and laminar flow resistance tube as the core components is designed. Through thermal insulation performance tests, flow resistance tests and measurement accuracy tests, combined with simulations, the effects of structural parameters and copper wire filling density on the performance of the isothermal container are analyzed, and these key parameters are optimized to improve the thermal insulation performance. A tubular laminar flow resistance tube composed of 304 stainless steel capillaries in parallel is designed. The influence mechanism of core parameters such as the number, radius, and length of laminar flow channels on its performance is studied, and the optimal parameter array is selected to optimize its performance. The experimental platform for measuring brake pressure change rate is constructed. By comparing the measurement curve of brake pressure change rate under simulation and experiment, the correctness and effectiveness of the pressure change rate measurement principle and the key components for electronically controlled pneumatic brakes of commercial vehicles are verified to meet engineering requirements.

## 1. Introduction

Pneumatic brakes have significant advantages in commercial vehicle brake systems owing to their high brake efficiency, high reliability and maintenance of brake capacity under transient failure of the power source. When the pneumatic brake system of a commercial vehicle is operated, there is pressure deviation or time deviation between the actual brake pressure response and the expected brake pressure response due to the problems of gas pressure fluctuation and transmission time delay. For the electronic pneumatic brake system applied to intelligent brakes, any response deviation may cause problems of vehicle brake safety, stability and ride comfort. Therefore, the pressure change rate considering both brake pressure response and brake time response should be used as the key control parameter of the electronically controlled pneumatic brake system to ensure that the brake pressure can change as expected. The real-time measurement of the brake pressure change rate is the basis of realizing accurate control of the electronic pneumatic brake system of commercial vehicles for intelligent braking.

Research on the vehicle brake pressure change rate mostly focuses on the field of hydraulic brake systems and lacks direct measurement methods. At present, it is based on pressure measurement and calculated indirectly through pressure differential. The data fluctuates greatly, and the calculation accuracy is low. Liang Chu et al. [1] obtained the relationship curve between the component parameters of ABS hydraulic control units and the brake pressure change rate based on the joint simulation platform of MATLAB and AMESim. In order to study the braking performance and the management of vehicles under the failure modes of the pneumatic ABS, Xiaohan Li et al. [2] established the co-simulation model of the pneumatic ABS of commercial vehicles based on AMESim and Simulink software. Jian Hu et al. [3] proposed the brake pressure change rate that comprehensively considers the brake pressure deviation and brake time deviation of electropneumatic brake systems for large-scale vehicles. The calculation model of the pneumatic brake pressure change rate was established by using MATLAB/Simulink and the accuracy was verified by experiments. Devika, K B et al. [4] developed a robust Proportional Integral Derivative (PID) controller using the Kharitonov theorem for pneumatic brakes. The efficacy of the brake controller when used for active safety system operation was investigated by conducting Hardware-in-Loop experiments.

Isothermal containers are often used to measure the flow characteristics of various pneumatic components, which can approximately solve the gas mass flow in the container. The international standards organization (ISO) has developed the draft ISO/DIS6385 standard [5], which uses sonic conductance C and critical pressure ratio B to represent the flow characteristics of pneumatic components. By using the pressure response of the fixed cavity, the flow characteristics of the pneumatic component can be measured in a short time and with low gas consumption. However, the pressure change inside the chamber will lead to a change in internal temperature, resulting in large measurement errors [6]. A single-phase flow of air in a channel bearing a circular cross-section with different arrangements of porous media was experimentally studied, and the results showed that fully filled channels of porous media exhibited the best heat transfer enhancement [7]. Tao Wang et al. [8] proposed a compensation method to reduce the impact of temperature change when measuring the flow characteristics of pneumatic components by an exhaust method. Based on the mathematical model of the quasi-isothermal container exhaust process, the temperature change compensation term expressed by exponential expression is used as a part of the flow equation. Yevtushenko A et al. [9] proposed an analytical numerical nonlinear model to study the temperature field and thermal stress of brake pads and discs during single braking at constant deceleration. Lihong Yang et al. [10] studied the influence of the distribution of fine copper wires in the container on the enhanced heat transfer based on the topological method. When the average porosity of fine copper wires is certain, the linear variable density method optimizes the distribution of fine copper wires in the container and verifies the effectiveness of the scheme. Wu SH et al. [11] proposed a topology optimization model for a transient heat conduction structure design. Numerous examples demonstrate that the optimized topological solutions of the transient heat conduction structure exhibit a remarkable transient effect.

Laminar flow elements are mostly used in laminar flow meters. Laminar flow state is a necessary condition for laminar flow meters. Bobovnik G et al. [12] studied the fluid-dynamic forces of laminar fluid flow in long vibrating pipes as well as in long curved pipes at rest. Additionally, the influence of the predicted values of the centrifugal correction factor on the onset of static and dynamic instabilities of pipes conveying fluid was also discussed. In order to solve the problem of measurement difficulty regarding the instantaneous flow of fuel and oxidant in pulse detonation engines, a rapid response flow measurement technology based on a laminar flow meter was proposed [13]. Jiang Y L [14] proposed a new method to calculate the weight function of electromagnetic flowmeters for gas–liquid two-phase flow. The simulation results show that this method can reasonably describe the influence of gas in the measured fluid on the output signal of the sensor, and the experimental results indirectly prove the rationality of this method.

In order to measure the pressure change rate of electronic pneumatic brakes of commercial vehicles in real time, control the pressure change rate during the brake process, and improve the safety, stability and ride comfort of vehicle brake systems, the rest of this paper is arranged as follows. In Section 2, based on Poiseuille’s law, the measurement principle of pressure change rate for electronically controlled pneumatic brakes of commercial vehicles is proposed, and the feasibility of the measurement principle is verified. In Section 3, according to the flow equation, state equation and motion equation, the simulation model of measurement-oriented pressure change rate for electronically controlled pneumatic brakes of commercial vehicles is established. In Section 4, the structural forms and parameters of the isothermal container and laminar flow resistance tube in the measurement of brake pressure change rate are designed and optimized. In Section 5, the simulation model of brake pressure change rate considering temperature change is established, and the brake pressure change rate measurement test platform is constructed. By comparing the results of the simulation and the test, the correctness and effectiveness of the measurement principle and key components of pressure change rate for electronically controlled pneumatic brakes of commercial vehicles are verified. The measurement principle of brake pressure change rate for commercial vehicles and key components designed in this paper will provide technical support for the precise control of the braking pressure for commercial vehicles and brake comfort control, which is of great significance to the development of intelligent safety technologies such as vehicle active safety, assisted driving and autonomous driving.

## 2. Measurement Principle and Verification of Pressure Change Rate for Electronically Controlled Pneumatic Brake of Commercial Vehicle Based on Poiseuille’s Law

### 2.1. Measurement Principle of Pressure Change Rate for Electronically Controlled Pneumatic Brake of Commercial Vehicle Based on Poiseuille’s Law

During the pneumatic braking of a commercial vehicle, due to the influence of supply pressure fluctuation, pressure transmission delay, and discontinuous switching action of the electrical control valve, there is pressure deviation or time deviation between the actual brake pressure response and the expected brake pressure response. These two deviations are called brake pressure deviation and brake time deviation, respectively, which are collectively referred to as brake pressure response deviation [3].

As shown in Formula (1), in order to ensure that the brake pressure changes as expected, taking the change of brake pressure per unit time, the brake pressure change rate, as the key control parameter and evaluation index are of great significance to the safety, stability and ride comfort for vehicle braking.

(1) $$\;\kappa =\frac{\Delta p}{\Delta t}=\frac{dp}{dt}$$

where, p is the output pressure of brake chamber, Pa.

Poiseuille’s law describes the relationship between the volume flow of viscous fluid $Q_{r}$ and the pressure difference at both ends of the horizontal circular tube under laminar flow:

(2) $$Q_{r}=\frac{p_{1}-p_{2}}{r_{rs}}$$

where, $p_{1}$ is the pressure at the left end of the circular tube, Pa; $p_{2}$ is the pressure at the right end of the circular tube, Pa; $r_{rs}$ is the flow resistance, $r_{rs}=\frac{8\mu_{r}L_{r}}{\pi r_{r}^{4}}$; $r_{r}$ is the radius of the circular tube,m; $\mu_{r}$ is the viscosity coefficient of fluid, Pa⋅s; $L_{r}$ is the length of the round pipe, m.

**Figure 1:**
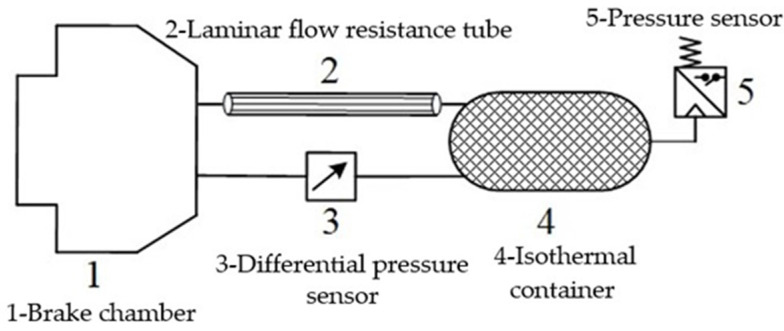
Schematic diagram of the measurement principle of pressure change rate for electronically controlled pneumatic brakes of a commercial vehicle.

Consisting of an isothermal container, laminar flow resistance tube, differential pressure sensor and pressure sensor, the schematic diagram of the measurement principle of pressure change rate for electronically controlled pneumatic brakes of a commercial vehicle is shown in Figure 1. The pressure in the brake chamber is $p_{b}$. The pressure difference between the brake chamber and the isothermal container $p_{d}$ is measured by the pressure difference sensor, and the pressure in the isothermal container $p_{i}$ is measured by the pressure sensor. Based on Poiseuille’s law and state equation, the relationship between pressure change rate $dp_{b}/dt$, differential pressure $p_{d}$ and pressure $p_{b}$ in the isothermal container can be obtained. In this paper, the pressure change rate in the isothermal container $dp_{i}/dt$ is taken as the measured value of the brake pressure change rate $dp_{b}/dt$ in the brake chamber, so as to realize the real-time measurement of the brake pressure change rate.

The full differential form of the state equation of the gas in the isothermal container is:

(3) $$V\frac{dp_{i}}{dt}+p_{i}\frac{dV}{dt}=GR\theta +mR\frac{d\theta }{dt}$$

Since the volume temperature of the container remains unchanged, the temperature in the container is the ambient temperature, which can be obtained from Formula (3):

(4) $$G=\frac{V}{R\theta_{a}}\frac{dp_{i}}{dt}$$

where, V is the volume of isothermal container, $m^{3}$; R is the gas constant,$\;J/\left(kg\cdot K\right)$; m is the mass of compressed gas in isothermal container,kg; θ is the temperature in the isothermal container, K; $\theta_{a}$ is the ambient temperature; G is the mass flow of gas, kg/s.

The laminar flow resistance tube is composed of n laminar flow channels with a radius of r and a length of L. The volume flow into the isothermal container is Q. It can be obtained from Formula (2):

(5) $$Q=\frac{\pi nr^{4}}{8\mu L}\left(p_{b}-p_{i}\right)$$

Since the temperature in the isothermal container remains unchanged, it can be obtained from the state equation of gas:

(6) $$\frac{p_{i}}{p_{a}}=\frac{\rho_{i}}{\rho_{a}}$$

Then the mass flow of the gas into the isothermal container is:

(7) $$G=\rho_{i}Q=\frac{\rho_{a}p_{i}\pi nr^{4}}{8p_{a}\mu L}\left(p_{b}-p_{i}\right)$$

Set the flow resistance coefficient of laminar flow resistance tube as:

(8) $$r_{s}=\frac{8p_{a}\mu L}{\rho_{a}\pi nr^{4}}$$

Equation (7) becomes:

(9) $$G=\frac{p_{i}}{r_{s}}p_{d}$$

where, $p_{a}$ is atmospheric pressure, Pa; $\rho_{a}$ is the atmospheric density, $kg/m^{3}$; $\rho_{i}$ is the density of compressed gas in isothermal container, $kg/m^{3}$; μ is the gas viscosity coefficient, Pa⋅s; $p_{b}$ is the output of the differential pressure sensor, Pa.

According to Equations (4) and (9), the measurement model of the brake pressure change rate under an isothermal condition is:

(10) $$\frac{dp_{i}}{dt}=\frac{p_{i}p_{d}}{r_{s}}\frac{R\theta_{a}}{V}$$

### 2.2. Laminar Flow Resistance Tube for Measuring Pressure Change Rate for Electronically Controlled Pneumatic Brake of Commercial Vehicle

The flow state of the gas in the laminar flow resistance tube can be determined by the Reynolds number Re. When Re is less than 2000, it can be proven that the gas in the laminar flow resistance tube is in laminar flow [15]. The calculation formula of Reynolds number is:

(11) Re=ρνdrμr

where, ρ is the density of fluid, $kg/m^{3}$; v is the velocity of fluid flow, m/s; $d_{r}$ is the inner diameter of circular pipe, m.

The response frequency of the pneumatic brake system can reach 20 Hz. In order to meet the real-time measurement demand of the pressure change rate for electronically controlled pneumatic brakes of a commercial vehicle, the response frequency of the laminar flow resistance tube is required to be higher than that of the pneumatic brake system, that is, the response time constant of the laminar flow resistance tube is less than 0.05 s.

Equation (10) can be transferred by Laplace transformation:

(12) $$sp_{b}-p_{b}\left(0\right)-sp_{d}+p_{d}(0)=\frac{1}{K_{a}}p_{d}p_{i}$$

where, $K_{a}$ is the gain coefficient, $K_{a}=\frac{r_{s}V}{R\theta_{a}}$.

Since at the equilibrium position $p_{d}(0)=0$, Equation (12) becomes:

(13) $$p_{d}=\frac{\frac{K_{a}}{p_{i}}}{1+\left(\frac{K_{a}}{p_{i}}\right)s}\left.\left\{sp_{b}-\right.p_{b}(0)\right\}$$

Since $sp_{b}-p_{b}(0)=\iota (\frac{dp_{b}}{dt})$, $p_{i}$ and $p_{d}$ can be measured by pressure sensor and differential pressure sensor, respectively, it can be obtained that:

(14) $$p_{do}=p_{i}p_{d}=\frac{K_{a}}{1+\left(\frac{K_{a}}{p_{i}}\right)s}\iota (\frac{dp_{b}}{dt})$$

where, the response time constant T is:

(15) $$T=\frac{K_{a}}{p_{i}}=\frac{8p_{a}\mu LV}{p_{i}\rho_{a}\pi nr^{4}R\theta_{a}}$$

The response time constant of the laminar flow resistance tube can be calculated from Equation (15). When the response time constant of the laminar flow resistance tube is less than 0.05 s, the real-time measurement demand of the brake pressure change rate can be met.

According to the pressure differentiator developed by Kawashima et al., this paper preliminarily sets the isothermal container volume $V=4.0\times 10^{-5}m^{3}$, number of laminar flow channels in the laminar flow resistance tube n=30, radius r=0.3 mm, length L=100 mm. In this paper, the test is carried out for the inflation process of the brake chamber. After differentiating the measured pressure in the brake chamber, it is shown that when the air supply pressure is 0.6 MPa and the sonic conductance is 2.2 m^3^/(s·bar), the maximum brake pressure change rate in the brake chamber can reach 2000 kPa/s. Gas constant R = 287 J/(kg·K), gas viscosity coefficient $\mu =18.1\times 10^{-6}Pa\cdot s$, the ambient temperature set to $\theta_{a}=293K$. Re=3720>2000 can be calculated from Equation (11), which does not meet the measurement requirements, so the structural parameters of laminar flow resistance tube need to be optimized.

When $p_{i}{=p}_{a}$, the value of response time constant T is the maximum. Upon substitution of V, n, r and L into Formula (15), it can be calculated that the maximum response time $T_{\max}=0.0076s$, which is less than the demand response time constant of 0.05 s, meeting the demand for real-time measurement of the brake pressure change rate.

### 2.3. Measurement Principle Verification of Pressure Change Rate for Electronically Controlled Pneumatic Brake of Commercial Vehicle

During inflation, the mass flow equation of air metal container is:

(16) $$G_{e}=\left\{\begin{array}{ll} \rho_{a}C_{e}p_{es}\sqrt{\frac{\theta_{a}}{\theta_{e}}} & \frac{p_{e}}{p_{es}}<b_{e}\\ \rho_{a}C_{e}p_{es}\sqrt{\frac{\theta_{a}}{\theta_{e}}}\sqrt{1-{\left(\frac{\frac{p_{e}}{p_{es}}-b_{e}}{1-b_{e}}\right)}^{2}} & \frac{p_{e}}{p_{es}}\geq b_{e} \end{array}\right.$$

where, $p_{es}$ is the air supply pressure of the air metal container, Pa; $\theta_{e}$ is the temperature in the air metal container, K; $C_{e}$ is the sonic conductance during the inflation of air metal container, $m^{3}/\left(s\cdot bar\right)$; $b_{e}$ is the critical pressure ratio of air metal container during inflation; $p_{e}$ is the pressure in the air metal container, Pa.

The gas state equation in the air metal container is:

(17) $$\frac{dp_{e}}{dt}=\frac{1}{V_{e}}\left(G_{e}R\theta_{e}+\frac{p_{e}V_{e}}{\theta_{e}}\frac{d\theta_{e}}{dt}\right)$$

(18) $$\frac{d\theta_{e}}{dt}=\frac{R\theta_{e}}{p_{e}V_{e}c_{v}}\left[c_{v}G_{e}\left(\theta_{a}-\theta_{e}\right)+R\theta_{a}G_{e}+h_{e}S_{e}\left(\theta_{a}-\theta_{e}\right)\right]$$

where, $G_{e}$ is the mass flow in the air metal container, kg/s; $V_{e}$ is the volume of air metal container, $m^{3}$; $c_{v}$ is the specific heat of fixed volume, $J/\left(kg\cdot K\right)$; $h_{e}$ is the heat exchange coefficient of air metal container, $W/\left(m^{2}\cdot K\right)$; $S_{e}$ is the heat exchange area of air metal container, $m^{2}$.

**Figure 2:**
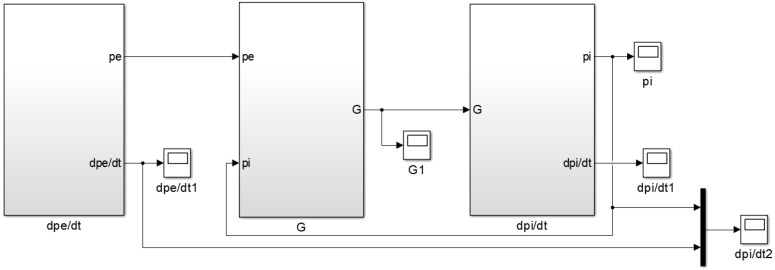
Simulation model of pressure change rate for the air metal container.

Based on the mass flow equation and gas state equation of the air metal container during inflation, combined with Equations (8)–(10), the simulation model of pressure change rate for air metal container is established, as shown in Figure 2.

**Figure 3:**
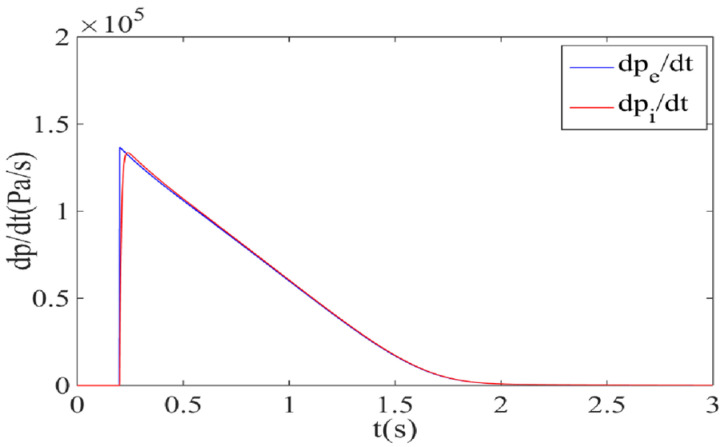
Calculation curve and measurement curve of pressure change rate of the air metal container by simulation.

When the air supply pressure is 0.2 MPa and the sonic conductance is 2.2 m^3^/(s·bar), the calculation curve and measurement curve of pressure change rate of the air metal container are obtained by simulation, as shown in Figure 3. It can be observed that the measurement curve of the pressure change rate is basically consistent with the calculation curve, which verifies the feasibility of the measurement principle of pressure change rate for electronic controlled pneumatic brakes of commercial vehicles.

## 3. Measurement Oriented Simulation Model of Pressure Change Rate for Electronically Controlled Pneumatic Brake of Commercial Vehicle

### 3.1. Simulation Model of Brake Chamber Inflation Process

Since the inflation and deflation processes are similar, only the simulation model for the inflation process of the brake chamber is established, and the simulation parameters of the brake chamber are shown in Table 1.

Based on the sonic conductance and critical pressure ratio, the mass flow equation of the brake chamber during inflation is as follows:

(19) $$G_{b}=\left\{\begin{array}{ll} \rho aCp_{bs}\sqrt{\frac{\theta a}{\theta_{b}}} & \frac{p_{b}}{p_{bs}}<b\\ \rho aCp_{bs}\sqrt{\frac{\theta a}{\theta_{b}}}\sqrt{1-{\left(\frac{\frac{p_{b}}{p_{bs}}-b}{1-b}\right)}^{2}} & \frac{p_{b}}{p_{bs}}\geq b \end{array}\right.$$

where, $p_{bs}$ is the gas pressure charged into the brake chamber, Pa; $\theta_{b}$ is the temperature in the brake chamber, K.

The gas state equation in the brake chamber during inflation is:

(20) $$\frac{dp_{b}}{dt}=\frac{1}{V_{b}}\left(G_{b}R\theta_{b}-p_{b}\frac{dV_{b}}{dt}+\frac{p_{b}V_{b}}{\theta_{b}}\frac{d\theta_{b}}{dt}\right)$$

(21) $$\frac{d\theta_{b}}{dt}=\frac{R\theta_{b}}{p_{b}V_{b}c_{v}}\left[c_{v}G_{b}\left(\theta_{a}-\theta_{b}\right)+R\theta_{a}G_{b}-p_{b}\frac{dV_{b}}{dt}+h_{b}S_{b}\left(\theta_{a}-\theta_{b}\right)\right]$$

where, $V_{b}$ is the volume of the brake chamber, $m^{3}$; $h_{b}$ is the heat exchange coefficient of the brake chamber, $W/\left(m^{2}\cdot K\right)$; $S_{b}$ is the heat exchange area of the brake chamber, $m^{2}$.

In Equations (20) and (21), the volume and effective cross-sectional area of the brake chamber can be calculated by the following formula:

(22) $$V_{b}=A_{b}\left(x_{b0}+\frac{\pi }{3}x_{b}\right)$$

(23) $$\frac{dV_{b}}{dt}=\frac{\pi A_{b}}{3}\frac{dx_{b}}{dt}$$

(24) $$A_{b}={\left(\frac{D_{b}}{2}\right)}^{2}+{\left(\frac{d_{b}}{2}\right)}^{2}+\frac{D_{b}d_{b}}{4}$$

where, $x_{b}$ is the displacement of piston disc, m; $x_{b0}$ is the initial displacement of the piston disc, m; $A_{b}$ is the effective cross-sectional area of the brake chamber, $m^{2}$; $D_{b}$ is the diameter of contact part between diaphragm and clamp, m; $d_{b}$ is the diameter of the contact interface between diaphragm and piston disc, m.

According to Newton’s second law, the motion equation is:

(25) $$\left(p_{b}-p_{a}\right)A_{bp}-m_{b}a_{b}-k_{b}x_{b}-F_{b}=0$$

where, $m_{b}$ is the total mass of the piston disc and push rod, kg; $A_{bp}$ is the effective bearing area of the piston disc, $m^{2}$; $a_{b}$ is the acceleration of the push rod, $m/s^{2}$; $F_{b}$ is the diaphragm force of the piston disc, N; $k_{b}$ is the spring elasticity coefficient, N/m.

**Figure 4:**
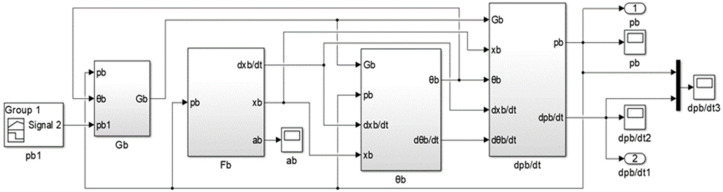
Simulation model of the brake chamber inflation process.

According to the flow equation, state equation and motion equation of the brake chamber, the simulation model of the brake chamber inflation process is established, as shown in Figure 4.

### 3.2. Simulation Model of Brake Pressure Change Rate Ignoring Temperature Change

**Figure 5:**
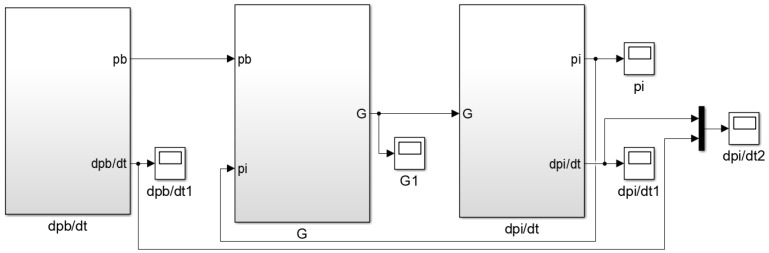
Simulation model of the brake pressure change rate ignoring temperature change.

Since the parameter design of the isothermal container has not been carried out, the temperature change in the isothermal container caused by an excessive pressure change rate cannot be considered, so the simulation model of the brake pressure change rate ignoring the temperature change is established firstly. Based on the simulation model of the brake chamber inflation process, the simulation model of the brake pressure change rate ignoring the temperature change is established, as shown in Figure 5.

## 4. Design and Analysis of Key Measuring Devices of Pressure Change Rate for Electronically Controlled Pneumatic Brake of Commercial Vehicle

### 4.1. Structural Parameter Design of Isothermal Container

In the process of inflation and deflation of the isothermal container, the internal heat transfer process mainly includes four processes: forced convection heat transfer process between compressed gas and the inner wall of the container, heat conduction process between the inner and outer walls of the container, natural convection heat transfer process between external air and the outer wall of the container, and convection heat transfer process between the compressed gas and copper wire. The first three heat exchange processes in the isothermal container have little effect on the fourth heat exchange process [16]. The heat exchange between the compressed gas in the container and the copper wire is the main heat exchange process.

**Figure 6:**
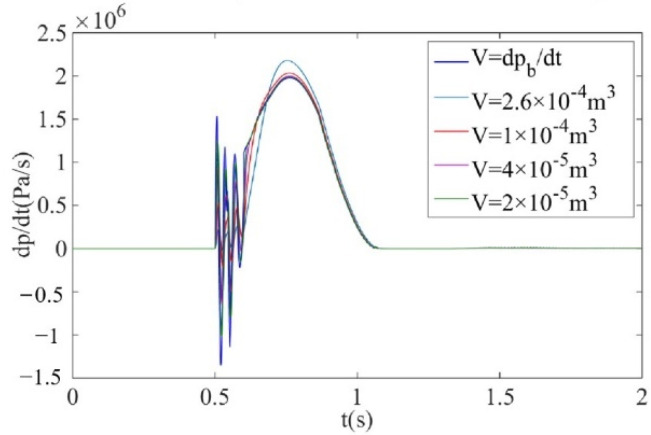
Simulation results of different isothermal container volumes.

**Figure 7:**
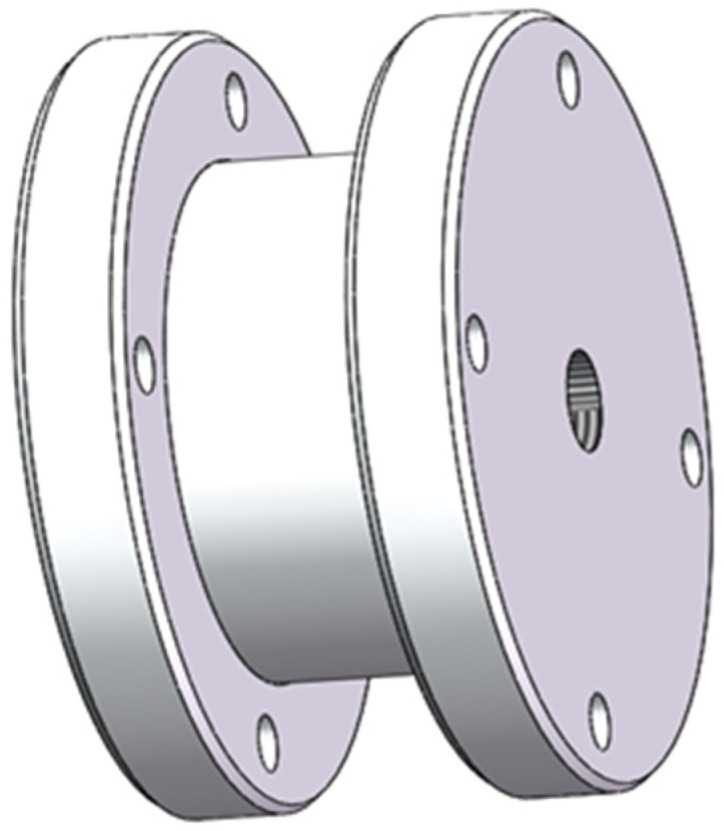
Three-dimensional diagram of the isothermal container.

It can be obtained from Equation (14) that the response time constant of the laminar flow resistance tube is only related to the volume of the isothermal container and is completely unrelated to its specific internal size. Therefore, the influence of the change in isothermal container volume on the change rate of the brake pressure is first analyzed, and its internal size is designed according to the actual needs. Based on the simulation model of the brake pressure change rate ignoring temperature change, combined with the preliminary key device structural parameters, the influence of isothermal container volume change on the measurement results of the brake pressure change rate is simulated and analyzed, so as to provide a reference basis for the isothermal container volume design. When the air supply pressure is 0.6 MPa and the sonic conductance is 2.2 m^3^/(s·bar), the performance of the isothermal container with a different volume is simulated and analyzed. The simulation results are shown in Figure 6. It can be observed that with the decrease of volume, the measurement curve of brake pressure change rate $dp_{i}/dt$ is closer to the calculation curve $dp_{b}/dt$, and when the volume of the isothermal container is less than $4\times 10^{-5}m^{3}$, there is almost no deviation between the measurement curve and the calculation curve of brake pressure change rate. The volume of the isothermal container should not be too small, which would cause the necessary pressure difference at both ends of the laminar flow resistance tube during the measurement process. Additionally, it is difficult to drill holes into the isothermal container. According to the above analysis, the volume of the isothermal container is $V=4\times 10^{-5}m^{3}$. Combined with the actual measurement requirements, the effective structure inside the isothermal container is designed as a cylinder with diameter d=50 mm and height h=20 mm. The three-dimensional diagram of the isothermal container is shown in Figure 7.

### 4.2. Analysis of Copper Wire Filling Density of Isothermal Container

In order to determine the filling density of copper wire when the isothermal container is used to measure the brake pressure change rate, the isothermal performance, flow resistance characteristics and measurement accuracy of isothermal container with different filling density of copper wire are studied in this paper. According to the different filling densities of copper wire, the isothermal containers are divided into four types: A, B, C and D, as shown in Table 2.

**Figure 8:**
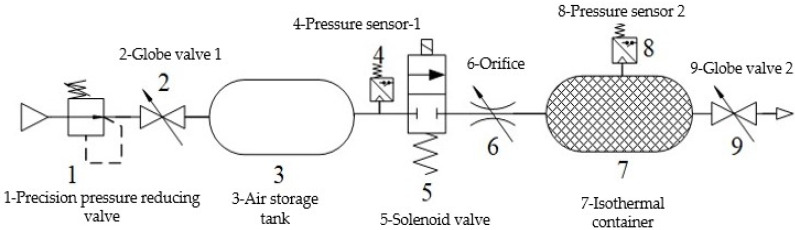
Block diagram of inflation test.

Since the process of inflation and deflation are similar, only the isothermal effect of the isothermal container during the inflation process under different copper wire filling densities is studied. A block diagram of the inflation test is shown in Figure 8.

Close the solenoid valve at any time during the inflation of the isothermal container and measure the pressure and temperature at this time. When the temperature of the gas in the container has restored to room temperature, measure again. Therefore, the temperature at any time in the inflation process can be calculated by the following formula:

(26) $$\frac{p_{i1}}{p_{i2}}=\frac{\theta_{i1}}{\theta_{i2}}$$

where, $p_{i1}$ is the gas pressure in the isothermal container at the moment when the solenoid valve is closed, Pa; $p_{i2}$ is the gas pressure in the container when the temperature of it changes to room temperature,  Pa; $\theta_{i1}$ is the temperature of gas in the isothermal container at the moment when the solenoid valve is closed, K; $\theta_{i2}$ is room temperature, K.

**Figure 9:**
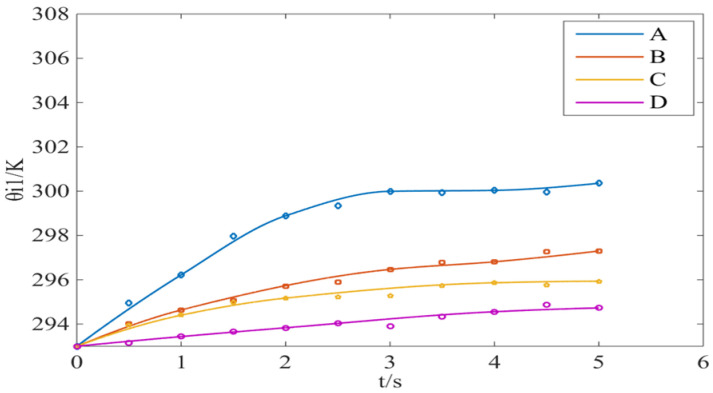
Temperature variation of the isothermal container inflation process under different filling densities.

In order to facilitate the test operation, the isothermal container with a volume of 1 L was used in the test. The temperature change of the isothermal container during inflation under different filling densities is shown in Figure 9. It can be observed that the higher the isothermal filling density of the copper wire in the container, the better the filling performance of the copper wire in the container.

**Figure 10:**
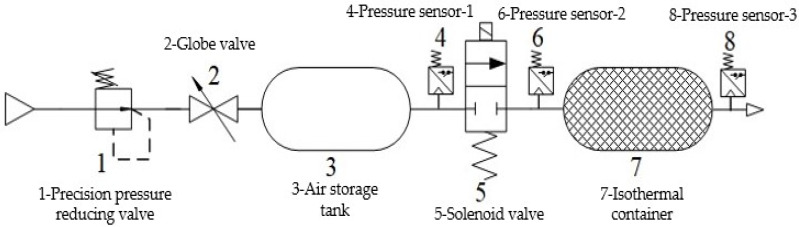
Schematic diagram of through flow resistance measurement test block diagram.

In this paper, the through flow resistance is measured to characterize the flow resistance of isothermal container, and its test block diagram is shown in Figure 10.

Measure the inlet and outlet pressure differences of four isothermal containers A–D ten times and obtain the average value. The test results are shown in Table 3. It can be observed that the greater the filling density of copper wire, the greater the through flow resistance.

**Figure 11:**
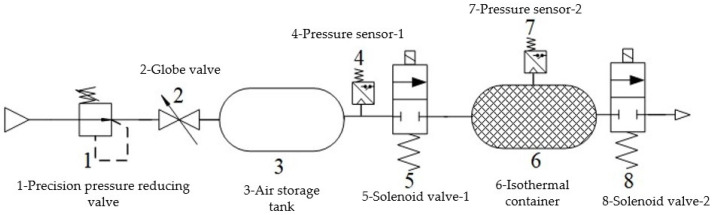
Schematic diagram of measuring sonic conductance of the solenoid valve by isothermal container venting method.

Change the filling density of copper wire in the isothermal container and measure the sonic conductance of the solenoid valve under different filling densities. Compare the measured value of sonic conductance with its ISO value and determine the filling density at which the change rate of brake pressure shows higher measurement accuracy. The measurement principle of the sonic conductance of the solenoid valve by isothermal container venting method is shown in Figure 11.

The pressure in the isothermal container $p_{i3}$ changes with time as follows:

(27) $$p_{i3}=p_{is}e^{-\frac{C_{i}R\rho_{a}\sqrt{293\theta_{a}}}{V_{i}}t}$$

where, $V_{i}$ is the volume of the isothermal container under the test conditions, $m^{3}$; $C_{i}$ is the sonic conductance of solenoid valve 8, $m^{3}/\left(s\cdot bar\right)$; $p_{is}$ is the initial pressure when the isothermal container is deflated, Pa.

The pressure change curve in the isothermal container during the venting process is measured by the test, the pressure value in the sonic stage is exponentially fitted with the corresponding time value, and then the sonic conductance of the solenoid valve is calculated by Equation (27). The results are shown in Table 4. When the filling density is 300 kg/m^3^, the accuracy of the isothermal container for measurement purposes is relatively high. Therefore, in this paper, the copper wire in the isothermal container is filled with a density of 300 kg/m^3^.

### 4.3. Structural Parameter Design of Laminar Flow Resistance Tube

In this paper, the laminar flow generator in the laminar flow resistance tube adopts a tubular structure, and its core design parameters are: the number n, radius r and length of laminar flow channels L and the inner diameter of the laminar flow resistance tube. Based on the simulation model of the brake pressure change rate ignoring temperature change, the influence of various parameters on the measurement results are simulated and analyzed in this paper, and a reference for core parameter selection of the laminar flow resistance tube is provided.

**Figure 12:**
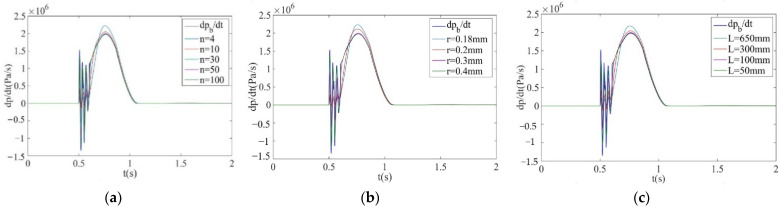
Simulation results of the pressure change rate under different channel numbers, radii and lengths. (**a**) Simulation results of different number of laminar flow channels. (**b**) Simulation results of different laminar flow channel radii. (**c**) Simulation results of different laminar flow channel lengths.

Combined with Formula (15) and the requirement that the response time constant is less than 0.05 s, when the air supply pressure is 0.6 MPa and the sonic conductance is 2.2$m^{3}/\left(s\cdot bar\right)$, change the number, radius and length of laminar flow channels, respectively, for simulation. The simulation results are shown in Figure 12.

It can be observed from Figure 12a that when the number of laminar flow channels n = 30, 50 or 100, the measurement curve is almost consistent with the calculation curve of the brake pressure change rate. Therefore, the number of laminar flow channels can be selected between 30 and 100. It can be observed from Figure 12b that when the radius of the laminar flow channel r = 0.3 mm or 0.4 mm, the deviation between the measurement curve and the calculation curve of the brake pressure change rate is relatively small, and when the laminar flow channel is too large, the necessary pressure difference cannot be formed, so the radius of laminar flow channel can be selected in the range of 0.3–0.4 mm. It can be obtained from Figure 12c that when the length of the laminar flow channel L = 100 mm or 50 mm, the deviation between the measurement curve and the calculation curve of the brake pressure change rate is small, and the length L is too short, which will lead to an insufficiently small resistance coefficient $r_{s}$ and will be unable to form the necessary differential pressure. Based on the above analysis, it is determined that the structural dimensions of the laminar flow resistance tube are: n = 54, r = 0.4 mm, L = 100 mm, and the inner diameter of the laminar flow resistance tube is 9 mm. The Reynolds number Re = 1550 and the response time constant $T_{max}$ = 0.0013 s which meet the measurement requirements.

## 5. Experimental Verification of Key Measuring Devices of Pressure Change Rate for Electronically Controlled Pneumatic Brake of Commercial Vehicle

### 5.1. Simulation Model of Brake Pressure Change Rate Considering Temperature Change

In the actual measurement, affected by the temperature change in the isothermal container, the actual measured pressure and differential pressure are also different from those measured in the isothermal container. The change of gas state in the isothermal container is as follows:

(28) $$\frac{{dp_{i}}^{\prime }}{dt}=\frac{G^{\prime }R\theta }{V}+\frac{m^{\prime }R}{V}\frac{d\theta }{dt}$$

(29) $$G^{\prime }=\frac{{pi}^{\prime }}{rs}\left(p_{b}-{pi}^{\prime }\right)$$

where, ${pi}^{\prime }$ is the actual measured pressure in isothermal container, Pa; θ is the actual measured temperature in isothermal container, K; $m^{\prime }$ is the actual measured mass of compressed gas in isothermal container, kg; $G^{\prime }$ is the actual measured mass flow through the laminar flow resistance tube, kg/s.

The actual measured output of differential pressure sensor is:

(30) $${pd}^{\prime }=p_{b}-{pi}^{\prime }$$

According to Equations (28)–(30), the measurement model of brake pressure change rate considering temperature change is:

(31) $$\frac{{dp_{i}}^{\prime }}{dt}=\frac{{pi}^{\prime }{pd}^{\prime }R\theta }{r_{s}V}+\frac{{pi}^{\prime }}{\theta }\frac{d\theta }{dt}$$

The heat transfer process in the isothermal container is complex. In order to more accurately reflect the thermodynamic process of inflation and deflation, it is necessary to comprehensively consider the influence of heat transfer. According to the first law of thermodynamics and the theory of variable mass thermodynamics, the energy conservation of the system can be expressed as:

(32) $$c_{v}m_{i}\frac{d\theta_{i}}{dt}+c_{v}G_{ic}\theta_{ic}-c_{v}G_{if}\theta_{i}=c_{p}G_{ic}\theta_{ic}-c_{p}G_{if}\theta_{i}-p_{ic}\frac{dV}{dt}+h_{i}S_{i}\left(\theta_{a}-\theta_{i}\right)$$

where, $h_{i}$ is the total heat transfer coefficient between the compressed gas and copper wire and container wall, $W/\left(m^{2}\cdot K\right)$; $S_{i}$ is the sum of the effective surface area of the copper wire and the container, $m^{2}$; $\theta_{i}$ is the temperature change in the container, K; $m_{i}$ is the mass of gas in the container, kg; $p_{ic}$ is the gas pressure in the container,Pa; $G_{ic}$ and $G_{if}$ are the mass flow of incoming and outgoing gas in the container,kg/s; $\theta_{ic}$ is the temperature of the gas flowing into the container, K; $c_{v}$ is the specific heat of fixed volume, $J/\left(kg\cdot K\right)$; $c_{p}$ is the specific heat at constant pressure, $J/\left(kg\cdot K\right)$.

Combined with Meyer’s formula $c_{p}=c_{v}+R$, the temperature of the incoming gas in the container is set as the ambient temperature, and the temperature response in the isothermal container during inflation is obtained as follows:

(33) $$\frac{d\theta_{i}}{dt}=\frac{R\theta_{i}}{c_{v}p_{ic}V}\left[G_{ic}\left(c_{p}\theta_{a}-c_{v}\theta_{i}\right)+h_{i}S_{i}\left(\theta_{a}-\theta_{i}\right)\right]$$

According to the calculation, the heat exchange area of copper wire $S_{c}$ is 0.108 m^2^ and the effective heat exchange area in the container is 0.004 m^2^. Therefore, the heat exchange area of the isothermal container is $S_{i}=S_{c}+S_{w}=0.112\;m^{2}$, and the average heat exchange coefficient $h_{i}=10W/\left(m^{2}\cdot K\right)$.

According to Formula (33), the temperature response in the container is:

(34) $$\frac{d\theta }{dt}=\frac{R\theta }{c_{v}{pi}^{\prime }V}\left[G^{\prime }\left(c_{p}\theta_{a}-c_{v}\theta \right)+h_{i}S_{i}\left(\theta_{a}-\theta \right)\right]$$

**Figure 13:**
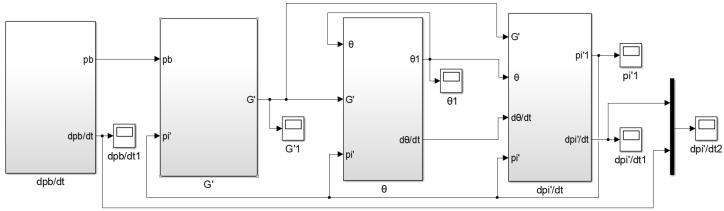
Simulation model of brake pressure change rate considering temperature change.

Based on the simulation model of the brake chamber with the inflation process, combined with Equations (29)–(31) and (34), the simulation model of the brake pressure change rate considering temperature change is established, as shown in Figure 13.

**Figure 14:**
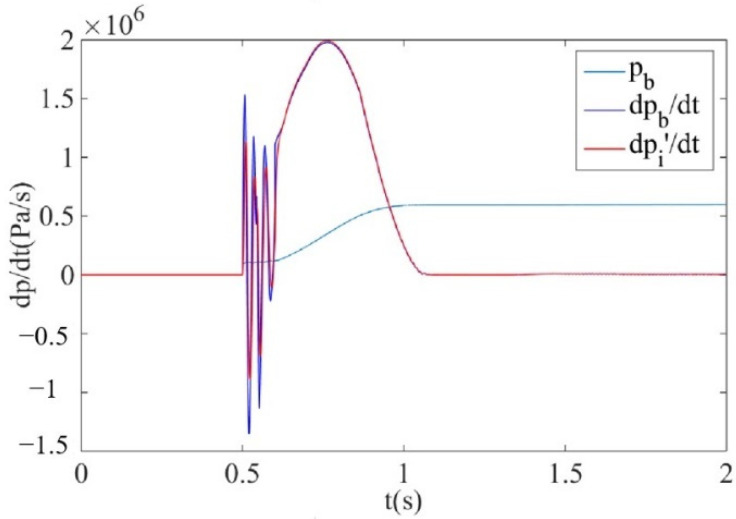
Simulation results of brake pressure change rate considering temperature change.

When the air supply pressure is 0.6 MPa and the sonic conductance is 2.2 m^3^/(s·bar), the calculation curve and measurement curve of the brake pressure change rate are obtained by simulation, as shown in Figure 14. It can be observed that the measurement curve of brake pressure change rate considering temperature change ${dp_{i}}^{\prime }/dt$ can be well consistent with the calculation curve of brake pressure change rate $dp_{b}/dt$. Therefore, the theoretical effectiveness of key devices and structural parameters of brake pressure change rate measurement are verified.

### 5.2. Pressure Change Rate Test Platform for Electronically Controlled Pneumatic Brake of Commercial Vehicle

**Figure 15:**
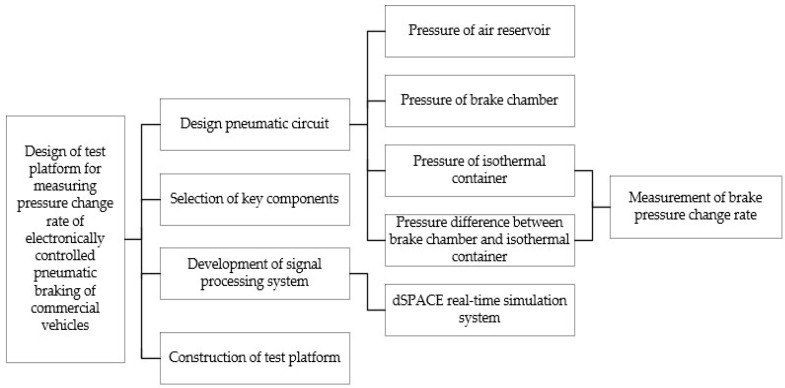
Overall design scheme of the brake pressure change rate measurement test platform.

**Figure 16:**
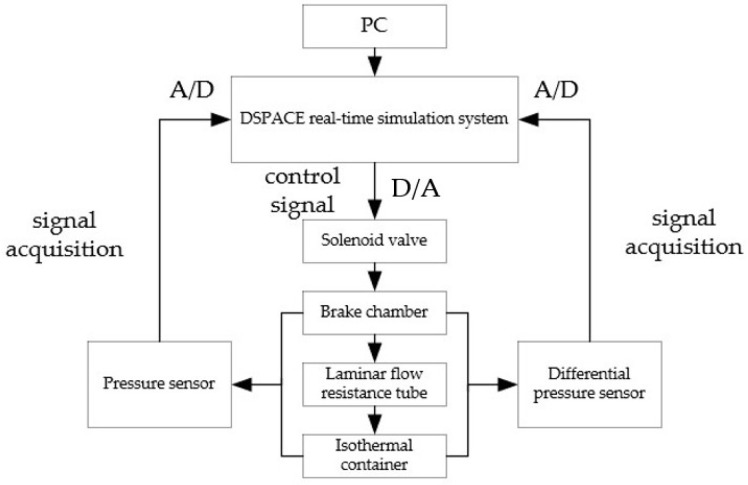
Overall structure of the brake pressure change rate measurement test platform.

Since the pressure measurement test of the brake chamber can be carried out in the real-time measurement circuit of the brake pressure change rate, the brake pressure change rate measurement test platform is the main design used in this paper. According to the test purpose and content, the overall design scheme of the brake pressure change rate measurement test platform is shown in Figure 15. The design of the brake pressure change rate measurement test platform includes pneumatic circuit design, key component selection, signal processing system development and test platform construction. The overall structure of the brake pressure change rate measurement test platform is designed, as shown in Figure 16.

**Figure 17:**
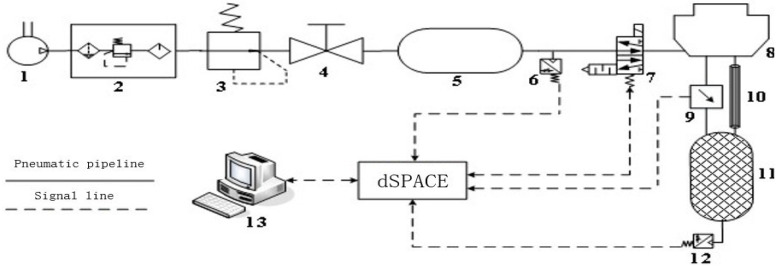
Block diagram of brake pressure change rate measurement. 1-Air source; 2-Pneumatic triplet; 3-Precision pressure reducing valve; 4-switch valve; 5-Air tank; 6,12-Pressure sensor; 7-Tow-position five-way solenoid valve; 8-Brake chamber; 9-Differential pressure sensor; 10-Laminar flow resistance tube; 11-Isothermal container; 13-PC.

**Figure 18:**
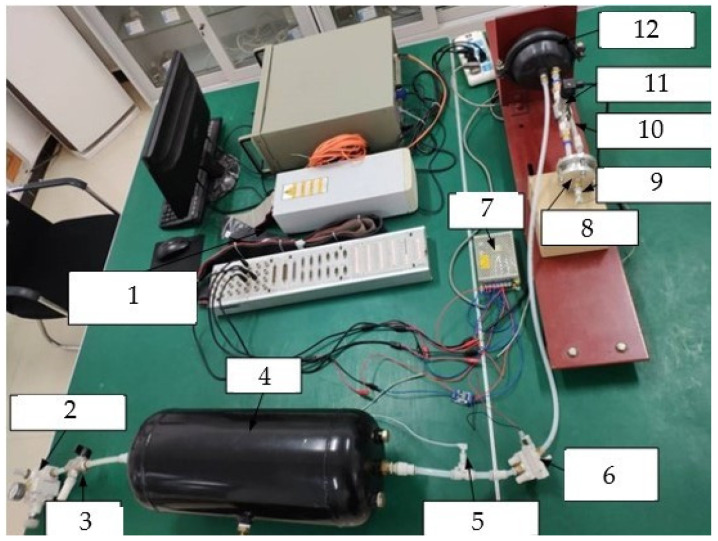
Brake pressure change rate measurement test platform. 1-dSPACE; 2-Precision pressure reducing valve; 3-On-off valve; 4-Air tank; 5-Pressure sensor; 6-Two-position five-way solenoid valve; 7-Power supply; 8-Isothermal container; 9-Pressure sensor; 10-Laminar flow resistance tube; 11-Differential pressure sensor; 12-Brake chamber.

According to the overall structure of the brake pressure change rate measurement test platform, the brake pressure change rate measurement circuit is designed, as shown in Figure 17. The brake pressure change rate measurement test platform adopts the dSPACE real-time simulation system for signal acquisition and processing. In this paper, an IPC-520 industrial computer is used to run dSPACE real-time measurements and control software, and the high-speed communication with DS1103 single board system was realized through Ethernet. The brake pressure change rate measurement test platform is constructed as shown in Figure 18.

### 5.3. Test Results Analysis of Key Measuring Devices of the Pressure Change Rate for Electronically Controlled Pneumatic Brake of Commercial Vehicle

**Figure 19:**
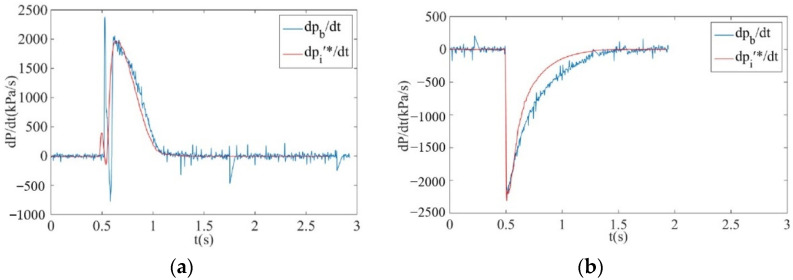
Comparison diagram of actual value and measured value of the pressure change rate during the inflation process and deflation process of the brake chamber. (**a**) Measurement curve of the brake pressure change rate during inflation process. (**b**) Measurement curve of the brake pressure change rate during deflation process.

During the inflation and deflation process of the brake chamber, for the inner chamber pressure data measured by the pressure sensor directly, the brake pressure change rate curve $dp_{b}/dt$ in the brake chamber is obtained by using the pressure change value in a certain step divided by the step. For the pressure and differential pressure data measured by isothermal container, laminar flow resistance tube, differential pressure sensor and pressure sensor, the temperature in the isothermal container was taken as the ambient temperature in Equation (31), and the measurement curve of brake pressure change rate was obtained ${dp_{i}}^{\prime *}/dt$. The results are shown in Figure 19.

**Figure 20:**
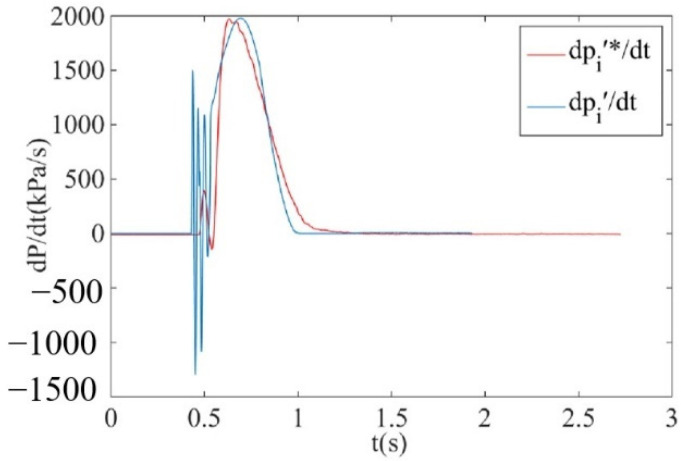
Measurement curve of the brake pressure change rate under simulation analysis and measurement test.

It can be observed from Figure 19 that due to the influence of the resolution of the pressure sensor, the brake pressure change rate curve obtained by the pressure sensor exhibits a large vibration, with an amplitude of about 100 KPa/s, but the brake pressure change rate curve can represent the real change trend of the brake pressure change rate in the brake chamber during inflation and deflation. The brake pressure change rate curve ${dp_{i}}^{\prime *}/dt$ measured by the isothermal container, laminar flow resistance tube, differential pressure sensor and pressure sensor can be better correlated with the brake pressure change rate curve $dp_{b}/dt$ obtained by the pressure sensor. Using the isothermal container, laminar flow resistance tube, differential pressure sensor and pressure sensor to measure the brake pressure change rate can not only allow real-time measurement of the brake pressure change rate, but there are also no large fluctuations in the measurement curve. It can be observed from Figure 20 that when comparing the measurement curve of brake pressure change rate under the simulation analysis and measurement test, there is a certain deviation between the simulation measurement curve and the actual measurement curve since there is a certain error between the simulation model of the brake chamber and the real object; however, the change trend of the measurement curve regarding the brake pressure change rate under the simulation analysis and measurement test remain basically consistent. Thus, the correctness and effectiveness of the measurement principle of the pressure change rate for electronically controlled pneumatic brakes of commercial vehicles and its key components are verified.

## 6. Conclusions

A measuring principle of gas pressure change rate is proposed. Based on Poiseuille’s theorem, key measuring elements such as an isothermal container and laminar flow resistance tube are designed and used to measure the change rate of braking pressure of commercial vehicles. It was shown that the simulation and experimental results were basically consistent, and the maximum error between them measured only 8%, which proves that this method can measure the pressure change rate effectively in real time. This is of great significance in promoting the development of intelligent safety technologies such as vehicle active safety, assisted driving and autonomous driving. Moreover, correcting the temperature change caused by variations in pressure and eliminating the small gap between parallel capillaries will further improve the measurement accuracy, which provides ideas and a theoretical basis for the optimization of this study.

## Figures and Tables

**Table 1:** Simulation parameters of brake chamber.

Parameter	Parameter Name	Value	Unit
ρ_a_	Atmospheric density	1.185	kg/m^3^
θ_a_	Ambient temperature	293	K
b	Critical pressure ratio	0.38	—
R	Gas constant	287	J/(kg·K)
c_v_	Specific heat of fixed product	718	J/(kg·K)
h_b_	Heat transfer coefficient	30	W/(m^2^·K)
x_b0_	Initial displacement of piston disc	0.0027	m
D_b_	Diameter of diaphragm in contact with clamp	0.21	m
d_b_	Contact diameter between diaphragm and piston disc	0.16	m
m_b_	Total mass of piston disc and push rod	0.53	kg
k_b_	Spring elasticity coefficient	3000	N/m
F_b0_	Initial deformation force of diaphragm	10	N
s_b_	Stroke of push rod	0.08	m

**Table 2:** Isothermal container with different copper wire filling density.

Type	A	B	C	D
Mass of filled copper wire per cubic meter (kg/m^3^)	100	200	300	400

**Table 3:** Through flow resistance of isothermal capacitor under different filling densities.

		Filling Density		
Type	A	B	C	D
Pressure difference (MPa)	0.0548	0.1123	0.2106	0.2327

**Table 4:** Measurement results of sonic conductance of the solenoid valve by isothermal container venting method.

Type	ISO		A	B	C	D
Sonic conductance	1.1	Measured value	1.1320	1.1069	1.1017	1.1059
C_i_ [m^3^/(s·bar)]		error (%)	2.91%	0.63%	0.15%	0.54%

## Nomenclature

**Symbol**	**Meaning**	**Unit**
$p_{b}$	The pressure in the brake chamber.	Pa
$p_{i}$	The pressure in the isothermal container.	Pa
$p_{d}$	The pressure difference between the brake chamber and the isothermal container.	Pa
Q	The volume flow into the isothermal container.	L/s
G	The gas flow into the isothermal container.	kg/s
V	Volume of isothermal container.	m^3^
R	Gas constant.	J/(kg·K)
m	Gas mass in isothermal container.	kg
θ	Temperature in isothermal container.	K
$\theta_{a}$	Ambient temperature.	K
$p_{a}$	Atmospheric pressure.	Pa
$\rho_{a}$	Atmospheric density.	kg/m^3^
μ	Gas viscosity coefficient.	Pa·s
n	Number of laminar flow channels.	—
r	Radius of laminar flow channel.	mm
L	Length of laminar flow channel.	mm
Re	Reynolds number.	—
T	Response time constant.	s
$c_{v}$	Specific heat at constant volume.	J/(kg·K)
$c_{p}$	Specific heat at constant pressure.	J/(kg·K)
$h_{i}$	Heat transfer coefficient of isothermal container.	W/(m^2^·K)
$S_{i}$	Heat exchange area of isothermal container.	m^2^
${pi}^{\prime }$	Pressure in isothermal container in actual measurement.	Pa
$m^{\prime }$	Gas mass in isothermal container in actual measurement.	kg
$G^{\prime }$	Mass flow in actual measurement.	kg/s
${pd}^{\prime }$	Actual measured differential pressure.	Pa

## References

[1] Chu L., Ou Y., Zhang Y.S. The mechanism study of ABS hydraulic control system brake pressure change rate. Proceedings of the International Conference on Computer Engineering and Technology.

[2] Li X., Zhao L., Zhou C., Li X., Li H. (2020). Pneumatic ABS Modeling and Failure Mode Analysis of Electromagnetic and Control Valves for Commercial Vehicles. Electronics.

[3] Hu J., Liu Y.D., Li X.L., Yang F., Li G.Y. Research on Large-Scale Vehicle of Electropneumatic Brake Pressure Change Rate for Braking Ride Comfort. Proceedings of the 20th COTA International Conference of Transportation Professionals.

[4] Devika K.B., Sridhar N., Patil H. (2020). Delay compensated pneumatic brake controller for heavy road vehicle active safety systems. Proc. Inst. Mech. Eng. C-J. Mech..

[5] (2013). Pneumatic Fluid Power—Components Using Compressible Fluids—Determination of Flow-Rate Characteristics.

[6] (2000). Pneumatic Fluid Power—Components Using Compressible Fluids—Determination of Flow-Rate Characteristics.

[7] Baragh S., Shokouhmand H., Ajarostaghi S. (2018). An experimental investigation on forced convection heat transfer of single-phase flow in a channel with different arrangements of porous media. Int. J. Therm. Sci..

[8] Wang T., Peng G.Z., Kagawa T. (2010). Determination of Flow-rate Characteristics for Pneumatic Components Using a Quasi-isothermal Tank with Temperature Compensation. Meas. Sci. Technol..

[9] Yevtushenko A., Kuciej M., Och E. (2018). Modeling of the temperature regime and stress state in the thermal sensitive pad-disk brake system. Adv. Mech. Eng..

[10] Yang L.H., Shen H.M. (2017). Effect of porous media distribution in isothermal vessel on enhanced heat conduction and isothermal properties. Chin. J. Mech. Eng..

[11] Wu S.H., Zhang Y.C., Liu S.T. (2019). Topology optimization for minimizing the maximum temperature of transient heat conduction structure. Struct. Multidiscip. Optim..

[12] Bobovnik G., Kutin J. (2018). Numerical study of the fluid-dynamic loading on pipes conveying fluid with a laminar velocity profile. J. Fluids Struct..

[13] Wang X.L., Zhang S.F., Chen Y.C. Research on Rapid Response Flow Measurement Technology Based on Laminar Flow Meter. Proceedings of the 9th International Conference on Mechanical and Aerospace Engineering (ICMAE).

[14] Jiang Y.L. (2020). Study on Weight Function Distribution of Hybrid Gas-Liquid Two-Phase Flow Electromagnetic Flowmeter. Sensors.

[15] Li F.B., Li Q., Wang D.X. (2017). Fluid Mechanics.

[16] Ye Q., Meng G.X., Xie W.H. (2007). Establishment and analysis of heat transfer model of isothermal container. Hydraul. Pneum..

[17] Bao H.W., Wang Z.Y., Liu Z.H. (2021). Study on Pressure Change Rate of the Automatic Pressure Regulating Valve in the Electronic-Controlled Pneumatic Braking System of Commercial Vehicle. Processes.

[18] Hu D.W., Li G.Y., Zhu G.M. (2020). A Control-Oriented Linear Parameter-Varying Model of a Commercial Vehicle Air Brake System. Appl. Sci..

[19] Drozdov E., Kuzmichev E. Improvement of Automatic Pneumatic Car Brakes. Proceedings of the 8th International Scientific Siberian Transport Forum (TransSiberia).

[20] Kaminski Z., Kulikowski K. (2021). Impact of a modified braking valve for static and dynamic characteristics of pneumatic braking systems of agricultural trailers. Int. J. Heavy Veh. Syst..

[21] Matveev V., Novikova Y., Popov G., Baturin O. Design and Operational Development a Pneumatic Braking System for a Gas-Turbine Units Test Bench. Proceedings of the ASME Turbine Technical Conference and Exposition (Turbo Expo).

